# Healthcare workers’ knowledge and risk perception regarding the first wave of COVID-19 in Khyber Pakhtunkhwa, Pakistan: an online cross-sectional survey

**DOI:** 10.1097/MS9.0000000000001916

**Published:** 2024-03-06

**Authors:** Iftikhar Ali, Zair Hassan, Arslan Rahat Ullah, Muhammad Noman Khan Wazir, Najma Fida, Muhammad Idrees Khan, Aysha Masood, Sayed Zulfiqar Ali Shah, Waqar Ali, Irfan Ullah, Adnan Ashraf, Arshad Hussain, Areeba Ahsan, Lina Hemmeda, Ghassan E. Mustafa Ahmed, Khabab Abbasher Hussien Mohamed Ahmed

**Affiliations:** a Paraplegic Center, Hayatabad; b Department of Cardiology, Lady Reading Hospital; c Department of Medicine & Allied, Northwest General Hospital & Research Centre; d Department of Physiology, Kabir Medical College; Departments of e Cardiology; f Pharmacy, Hayatabad Medical Complex; Departments ofg Pharmacy; h Social Work, University of Peshawar; i Kabir Medical College, Gandhara University, Peshawar, Khyber Pakhtunkhwa; j Foundation university school of health sciences, Islamabad, Pakistan; k Department of Thoracic Medicine, Royal Bournemouth Hospital, Castle Ln E, Bournemouth, UK; l Department of Rehabilitation Medicine, Tongji Hospital, Tongji Medical College, Huazhong University of Science and Technology, Wuhan, People’s Republic of China; m Undergraduate Research Organizations, Dhaka, Bangladesh; n Faculty of Medicine, University of Khartoum, Khartoum, Sudan

**Keywords:** coronavirus disease 2019(MeSH), COVID-19 (MeSH), healthcare workers (MeSH), knowledge (non-MeSH), Pakistan (Non-MeSH), pandemic (MeSH), risk perception (Non-MeSH)

## Abstract

**Background:**

Increased COVID-19 transmission among the populace may be caused by healthcare workers (HCWs) who lack knowledge, awareness, and good preventive practices. Additionally, it may cause elevated stress levels, anxiety, poor medical judgement, and situational overestimation.

**Objectives:**

The present survey aimed to assess knowledge and risk perception regarding COVID-19 among HCWs in Khyber Pakhtunkhwa (KP), Pakistan.

**Methodology:**

A web-based online, pre-tested questionnaire comprising 26 items was circulated via social media in April 2020 amongst HCWs in major tertiary care facilities in KP.

**Results:**

The study’s results, revealing both the commendable knowledge levels among HCWs about COVID-19 and their heightened risk perception, highlight the critical need for targeted interventions to address the potential impact on self-protective behaviour and mental health within this vital workforce. This insight is important for designing strategies that not only enhance HCWs’ well-being but also ensure the continued effectiveness of healthcare delivery during pandemics. The percentage mean score (PMS) of COVID-19 knowledge was 85.14±10.82. Male HCWs and those with an age older than or equal to 32 years demonstrated a higher knowledge score (85.62±11.08; *P*=0.032 and 87.59±7.33, *P*=0.021, respectively). About 76% of HCWs feared contracting COVID-19. Nearly 82% of respondents were mentally preoccupied with the pandemic and also terrified of it. ‘Of these, 81% were nurses, 87% had a job experience of 6–8 years and 54.45% were frontline workers. Feelings of panic and concern about the pandemic were found to be more in HCWs who were physicians above the age of 32, and who had 3–5 years of work experience. HCWs’ overall risk perception was found to be significantly different between males (7.04±2.26) and females (8.01±1.97), job experience of 6–10 years (8.04±177) with 3–5 years and younger than or equal to 2 years job experience (7.18±2.43,6.93±2.22), respectively, and between frontline HCWs (7.50±2.10) and non-frontline HCWs (6.84±2.40).

**Conclusion:**

HCWs demonstrated good knowledge about COVID-19. As the risk perception of COVID-19 among HCWs is high, it can raise concerns about their self-protective behaviour, and mental health. These issues need to be addressed.

## Introduction

Highlights

- Healthcare workers (HCWs) who lack knowledge, awareness, and appropriate preventive practices may be the source of increased COVID-19 transmission among the general public. It may also result in increased worry, tension, poor medical judgement, and overestimation of the circumstances.
- The current study sought to evaluate HCWs’ perceptions of risk and level of knowledge of COVID-19 in Khyber Pakhtunkhwa (KP), Pakistan.
- In April 2020 HCWs at significant tertiary care facilities in KP were given access to a web-based, pre-tested questionnaire consisting of 26 items via social media.
- HCWs showed a strong understanding of COVID-19. Regarding HCWs’ self-protective conduct and mental health, there may be concerns due to their elevated risk perception of COVID-19. We must take action on these concerns.

The novel coronavirus first surfaced in the last days of December 2019 in China. This novel virus strain was termed 2019-nCoV by the “International Committee on Taxonomy of Viruses”. Later on, it was given the name “Severe Acute Respiratory Syndrome, Coronavirus-2 (SARS-CoV-2)”. The WHO on 11 February 2020, proposed a new title for SARS-CoV-2—COVID-19^1,2^. Due to the rapid transmission of this virus from its epicentre to several countries, WHO on 30 January 2020 declared it a Public Health Emergency of International Concern (PHEIC)^3^.

The escalating outbreak of COVID-19 shook the nations worldwide, affecting millions of people in ~216 countries (including areas or other territories). At the time of writing, there had been a total of 6 799 713 confirmed cases across the globe, including 397 388 deaths^4^. In Pakistan, the first case was reported in Karachi on 26 February and by midnight of 7 June 2020, the country had reported a total of 98 943 cases and 2002 related deaths^4^. The pandemic caused by the COVID-19 virus has affected every aspect of society, including healthcare, economics, and political effects^5^.

For instance, in India, the high transmissibility and infection rate of SARS-CoV-2 during the second wave led to viral spread in the asymptomatic population, especially those who stayed inside, contributing to an increase in positivity and death^6^.

SARS-CoV-2 is considered to be of zoonotic origin. Henipaviruses are a category of viruses that can be transmitted amongst bats as well as other animal species and humans. The primary viral organisms within this group encompass the Nipah virus (NiV) and Hendra virus (HeV), as well as the former SARS-CoV-2 pandemic^7,8^.

This supports the interconnection theory between animals and human health, as in a WHO report where more than fifty instances of monkeypox infection have recently been confirmed and published in the UK, posing a serious public health issue amid the COVID-19 pandemic^9^. In addition, the most recent COVID-19, monkeypox, poliovirus, and respiratory syncytial virus (RSV) outbreaks have all provided additional evidence of such interconnection^10^.

The ambiguity of SARS-CoV-2 transmission lies in the uncertainty about its mode of transmission, whether it is droplet or airborne transmission is continuing to be a topic of debate^11^.

Several evidence suggest that SARS-CoV-2 is primarily, transmitted from human to human via respiratory droplets, sneezing, coughing, and direct contact with infected persons, and has an incubation period of 2–14 days. It is also believed that people with SARS-CoV-2 with no symptoms can transmit the disease, ultimately substantiating the risk. To date, no effective treatment is available and elderly people, patients with chronic medical conditions and healthcare workers (HCWs) are more likely to get the infection. The clinical manifestations include fever, shortness of breath, myalgia, dry cough and fatigue^12^. Severe cases are usually characterized by acute respiratory distress syndrome, metabolic acidosis, sepsis, and coagulation disorders^13,14^. Public health measures such as social distancing, managed isolation, hand hygiene and droplet precautions are considered to be the main preventive options. On the other hand, herd immunity to SARS-CoV-2 can be achieved through recently developed vaccination^15^. It is worth noting that the scientific community has made significant efforts to encounter various infectious disease outbreaks in the last three decades, such as SARS, MERS, SARS-CoV-2 and mpox. Of these efforts, the 18^th^ Heads of State and Government Summit of the Group of 20 (G20) took place on 9 and 10 September 2023 in New Delhi, India. 3 which announced a new Global Initiative on Digital Health (GIDH), a joint effort between the WHO and the G20 India presidency^16,17^.

In addition, artificial intelligence such as Chatgpt can be employed to provide real-time information on disease outbreaks and health risks in various destinations, as well as assist travellers in understanding the necessary vaccinations and medications they may need for their trips^18^. Moreover, AI-based applications and other mathematical techniques have been employed in research opportunities for different applications regarding lung characterization in several pathologies^19^.

The pandemic wreaks havoc across the region and escalates fear and apprehension among the general masses as the situation continues to worsen. HCWs are no exception^20^, as they are the first respondents to come into contact with patients and are at risk of being exposed to SARS-CoV-2 in the hospital setting, which long hours, psychological stress, fatigue, and overcrowded conditions. A study in China showed 29% transmission to HCWs^21^. A patient in Wuhan, who was planning to undergo surgery, had infected 14 HCWs before fever was initiated^22^. According to a recent report, thousands of HCWs have contracted COVID-19^21^. As of 30 May 2020, the virus had affected 1904 HCWs with 70 deaths in Pakistan since the outbreak emerged^23^.

Lack of knowledge, and awareness and poor preventive practices among HCWs can lead to increased transmission of COVID-19 amongst this population^24^. It can also lead to an increase in stress levels, anxiety, impaired medical judgement, and overestimating situations. A study on knowledge and risk perception toward COVID-19 amongst HCWs is therefore necessary since the infection curve among HCWs is on the rise in Pakistani healthcare institutions. Knowledge and risk perception are the core components that need careful evaluation in this regard. This study aims to assess the knowledge and risk perception of COVID-19 among HCWs in Khyber Pakhtunkhwa, Pakistan, with a specific focus on understanding the potential implications of their awareness levels and perceived risks on self-protective behaviour and mental health.

## Methods

### Study design and participants

A web-based survey targeting HCWs was carried out from 12 to 22 April 2020 in all major tertiary care hospitals of KP. Keeping in view the specific infection prevention recommendations during the pandemic (social distancing and contact precautions), and that the majority of HCWs have internet access, we considered using an online platform to collect data.

### Sampling method

The participants were selected using a stratified sampling method. We categorized healthcare workers based on their roles (e.g. physicians, nurses, support staff) to ensure a diverse representation.

### Inclusion criteria for HCWs

We included healthcare workers from major tertiary care hospitals in Khyber Pakhtunkhwa, encompassing various roles such as physicians, nurses, and support staff. This inclusive approach aimed to capture a comprehensive view of knowledge and risk perception across different healthcare roles.

### Sample size

The Raosoft calculator was used to determine the sample size of our study, based on the population of HCWs. Considering a response distribution of 50%, CI of 95%, standard deviation of 1.96 and error margin of 5%. This resulted in a sample size of 381. Furthermore, 10% (*n*=37) was considered for any error in filling out the questionnaire. Four hundred and fourteen were taken as the final sample size for our study. However, a total of 468 HCWs responded and completed the survey. Based on valid responses 371 of these were considered for the final analysis. The HCWs excluded from our study were mainly either abroad or out of the province at the time of the study.

### Response rate

Out of the 468 HCWs contacted, 371 actively participated in the survey, resulting in a response rate of 79.06%. This response rate suggests a high level of engagement among the healthcare workers.

### Data collection

A self-administered questionnaire through the Google Forms platform was circulated via social media (WhatsApp and Facebook). The participants, receiving the online questionnaire, were requested to confirm their willingness to participate in the survey. Participation in the survey was purely voluntary, and respondents received no compensation. Ethical considerations were paramount in this research project. Participants were provided with informed consent, and the study design and implementation adhered to ethical standards. The anonymity and confidentiality of respondents were meticulously preserved, and the research protocol received approval from relevant institutional ethics committees.

### Data collection tool

The study tool was designed based on the existing literature published on SARS-CoV-2^21–23^. The validity of the questionnaire was established by panel experts including a pharmacist with qualifications and experience in public health, an epidemiologist, an infectious disease specialist and a physician. For understanding and harmony, changes were made in the order of items, along with some language modifications. Cronbach’s Alpha (α=0.83) was calculated by piloting the questionnaire in 35 HCWs before the actual questionnaire was circulated.

The final version of the tool included 26 items based on participant characteristics, information sources, knowledge, and risk perception towards COVID-19. The questionnaire consisted of 3 parts:

- The first section consisted of 6 items (participant characteristics) including age, gender, profession and experience in years, hospital affiliation and whether the participant was a frontline HCW.
- The second section (knowledge part) comprised 15 items. The framework of these items was based on a tool used in an earlier publication on Middle Eastern Respiratory Syndrome and on recently published studies on COVID-19^22,24^. These questions were answered in a ‘true’, ‘false’ and ‘I don’t know’ format. A correct answer was allotted 1 point while an uncorrected “I don’t know” answer was assigned 0 points. The knowledge mean score was converted into a percentage mean score (PMS).
- The third section consisted of 5 items related to risk perception and source of information. Three statements regarding risk perception were provided and the responses were recorded as either ‘yes’ or ‘no’, whereas overall risk perception was assessed using a visual analogue scale (0 not risky at all, and 10=very risky).

### Statistical analysis

SPSS Version 21 was used for analysis. The frequencies (%) and mean (SD) were calculated for categorical and numerical variables, respectively. Normality was assessed using Kolmogorov-Smirnov statistics. χ^2^ or the Fischer exact test was used for categorical variables that were appropriate. PMS of COVID-19 knowledge score and overall, VAS score were tested using nonparametric statistics such as the Mann–Whitney test and Kruskal–Walli’s test. A *P* less than 0.05 was taken as statistically significant for all tests.

This work has been reported in line with the STROCSS criteria^25^.

## Results

Of the total, 287 (77.36%) were male. The mean (SD) age of the HCWs was 29.10±5.32 years and the majority 227 (61.2%) of the HCWs were in the age group of 26–32 years. By profession, the highest numbers of respondents were nurses who were 122 (32.9%) of the sample followed by resident medical officers 83 (22.4%) and 35 (14.3%) were house officers. About forty-six per cent of HCWs had younger than or equal to 2 years of job experience. The details are given in Table 1.

**Table 1:** Background characteristics of the healthcare workers and outcome variable

Variable	*N* (%)
Age groups	
20–25 years	86 (23.2)
26–32 years	227 (61.2)
>32 y	58 (15.6)
Sex	
Female	84 (22.64)
Male	287 (77.36)
Profession	
Physician	9 (2.4)
Residents Medical Officer	83 (22.4)
Medical Officer	46 (12.4)
House Officer	53 (14.3)
Pharmacist	26 (7.0)
Nurses	122 (32.9)
Medical Technician	32 (8.6)
Job experience (year)	
≤2	171 (46.09)
3–5	116 (31.27)
6–10	84 (22.64)
Hospitals	
Ayub Teaching Hospital[ATH], Abbottabad	21 (5.66)
Hayatabad Medical Complex[HMC], Peshawar	105 (28.30)
Khyber Teaching Hospital[KTH], Peshawar	56 (15.09
Lady Reading Hospital[LRH], Peshawar	72 (19.41)
Mardan Medical Complex[MMC], Mardan	18 (4.85)
Northwest general Hospital & research centre[NWGH], Peshawar	24 (6.47)
Rehman Medical Institute[RMI], Peshawar	31 (8.36)
Saidu Teaching Hospital[STH], Swat	44 (11.86)
Frontline HCW	
Yes	236 (63.61)
No	135 (36.39)
Risk perception COVID-19 is a significant public health problem in Pakistan	
Yes	330 (88.9)
No	41 (11.1)
Do you feel distressed and panicked regarding COVID-19 infection in Pakistan?	
Yes	304 (81.9)
No	67 (18.1)
There is a high chance for you to be infected? [God forbid]	
Yes	283 (76.3)
No	88 (23.7)

HCW, healthcare worker; N, frequency.

The percentage mean value of COVID-19 knowledge score was 85.14±10.82 while the average correct answers were 85.19%. The question the highest number of respondents got wrong was about the usual incubation period for coronavirus SARS-CoV-2 232 (62.53%). This was followed by 127 (34.2%) of HCWs who did not know the distance required for airborne transmission of the virus. About forty percent of HCWs did not know the clinical situations when an N95 mask was required (such as during suctioning, intubation, bronchoscopy and cardiopulmonary resuscitation). The details of the incorrect responses in serial order are given in Table 2.

**Table 2:** Number of incorrect answers to individual COVID-19 Knowledge items

S. no	Items	*N* (%)
1.	COVID-19 is caused by SARS-CoV-2.(True)	69 (18.60)
2.	The first case of COVID-19 was surfaced in Wuhan City (China). (True)	07 (01.89)
3.	The common symptoms of COVID-19 include fever, dry cough, dyspnoea, fatigue and myalgia. (True)	05 (01.35)
4.	COVID-19 can be spread from contact with contaminated objects/surfaces such as doors, tables, knobs and mobile phones etc.(True)	20 (05.39)
5.	COVID-19 incubation period is 05-21 days.(False)	232 (62.53)^a^
6.	The COVID-19 spreads primarily via coughing, sneezing or respiratory droplets.(True)	29 (07.82)
7.	COVID-19 can cause pneumonia, respiratory failure, and death.(True)	17 (4.58)
8.	Treatment is not available for COVD-19, but early symptomatic treatment can also help most of the patients recover from the infection.(True)	14 (3.77)
9.	COVID-19 does not always progress to the severe level. Only the elderly, those with chronic conditions (diabetes, hypertension, cardiovascular disease, cancer, asthma, etc.), and those who are obese are vulnerable. (True)	29 (7.82)
10.	Managed Isolation and treatment of people who are infected with the COVID-19 are effective ways to reduce the transmission of the disease.(True)	16 (4.31)
11.	People who have contact with someone infected with the COVID-19 virus should be immediately isolated in a proper place. In general, the observation period is 14 days.(True)	10 (2.70)
12.	Hand hygiene (washing hands, using sanitizers, etc.), covering the nose and mouth while coughing and sneezing, and avoiding contact with sick people will all help to prevent the spread of COVID-19.(True)	4 (1.08)
13.	Only during suctioning, intubation, bronchoscopy and cardiopulmonary resuscitation, Healthcare professionals have to wear N95 mask.(True)	148 (39.89)^a^
14.	Approximately how much distance in feet do you think that the coronavirus can travel through the air to transmit the infection from one person to another?(6 feet)	127 (34.23)^a^
15.	People with COVID-19 can transmit the infection only after their symptoms appear. (False)	97 (26.15)

N, frequency.

a High incorrect percentages.

**Figure 1:**
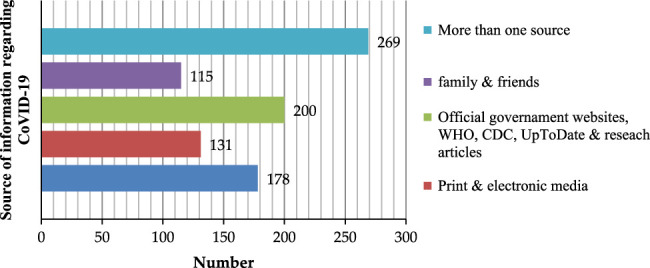
Information sources stated by healthcare workers. CDC, Center for Disease Control and Prevention.

Most HCWs in our study would look to official government websites, the WHO and the Center for Disease Control and Prevention (CDC) for COVID-19-related information 200 (53.9%). Nearly seventy-three percent of respondents would refer to the resource UpToDate and research articles, whereas 47.97% (178) would obtain their information regarding COVID-19 via social media. Figure 1 illustrates the sources HCWs got their COVID-19-related information from.

While evaluating the HCWs’ knowledge about COVID-19, compared to the age groups 20–25 years (PMS, 81.09±17.05) and 26–32 years (PMS, 86.05±7.77), HCWs aged older than or equal to 32 years had the highest knowledge score (PMS, 87.59±7.33) and the differences were statistically significant (*P*=0.021). Male HCWs obtained a high score (PMS 85.62±11.08; *P*=0.032) compared to females (PMS 83.49±9.79). Moreover, HCWs with job experience of 3-5 years had better knowledge (PMS 86.38±7.38), compared to HCWs with younger than or equal to 2 years’ (PMS 84.05±13.36) and 6–10 years (PMS, 85.63±8.75) experience respectively but the percentage mean score did not differ significantly. HCWs whose primary source of COVID-19-related knowledge was from family and friends had a lower mean score (82.26±15.39; *P*=0.016) compared to those who did not rely on this source (86.43±7.66) and the difference was statistically significant. Additionally, HCWs whose primary source of information was from official government websites (WHO, Up-to-date, and research articles) possessed better knowledge but did not differ significantly from those who did not rely on these resources for knowledge. (85.60±8.17 vs. 84.60±13.28) (Table 3).

**Table 3:** Knowledge scores across hcw different characteristics.

Variable	Mean±SD	*P*
Age groups (year)		
20–25	81.09±17.05	0.021
26–32	86.05±7.77	
>32	87.59±7.33	
Sex		
Male	85.62±11.08	0.032
Female	83.49±9.79	
Profession		
Physician	87.41±7.03	0.381
Residents Medical Officer	87.07±7.24	
Medical Officer	85.80±7.38	
House Officer	85.28±7.32	
Pharmacist	86.67±8.84	
Nurses	84.04±13.86	
Medical Technician	81.25±15.19	
Job experience (year)		
≤2	84.05±13.36	0.563
3–5	86.38±7.38	
6–10	85.63±8.75	
Frontline HCW		
Yes	85.59±1127	0.066
No	84.34±9.98	
Source of information regarding COVID-19		
Social media	84.98±9.04	0.400
Print and electronic media	83.61±12.68	0.057
Official government websites, WHO, CDC, UpToDate etc	85.60±8.17	0.592
Family and friends	82.26±15.39	0.016
More than one source	84.71±11.76	0.428

CDC, Center for Disease Control and Prevention.

Regarding the HCWs’ risk perception, a majority 330 (88.9%) of the HCWs agreed that COVID-19 is a significant public health problem in Pakistan. About 76.3% of the HCWs were paranoid with the contemplation of contracting COVID-19. Nearly 82% of the HCWs were preoccupied and terrified regarding the COVID-19 epidemic in Pakistan (Table 1). VAS was used to assess the overall perceived risk of COVID-19; the mean value of overall risk perception was 7.26±2.23.

More than 93% of the HCWs in the age groups range between 26 and 32 years, and 96.2% of house officers, believed that the current COVID-19 pandemic is a significant public health concern in Pakistan; the associations were statistically significant (*P*<0.05) among the age groups and professions. Likewise, 93.1% of HCWs below 32 years of age and physicians with a job experience of 3–5 years were concerned and terrified about the COVID-19 pandemic in Pakistan(*P*<0.05). About 81.1% of the nurses (*P*=0.029) and those with a job experience of 6–10 years 86.9% (*P*=0.012) were paranoid about the contemplation of contracting COVID-19. Furthermore, those HCWs who were in direct contact with patients believed there was a high chance for them to get infected, and this was statistically significant. Statistically, a significant association was noted between the statements “COVID-19 is a significant public health problem in Pakistan “and HCWs who rely on the information from print and electronic media or inquire about and discuss COVID-19-related aspects with family and friends. Similarly, those who relied on or received the information or discussed the topics with family and friends regarding COVID-19 showed the significance of the statement “There is a high chance for you to be infected”.

Overall HCWs’ perceived risk of COVID-19 was assessed, and a considerable mean difference was observed between males (7.04±2.26) and females (8.01±1.97). Additionally, among the HCWs the VAS score did differ significantly with HCWs who had a job experience of 6–10 years securing rather a higher value (8.04±177) than those with a job experience of 3–5 years (7.18±2.43) or less than or equal to 2 years job experience (6.93±2.22). Similarly, a significant mean difference was noted between frontline HCWs (7.50±2.10) and non-frontline HCWs (6.84±2.40).

Furthermore, the overall perceived risk score was higher for those who had received the information or discussed COVID-19 topics with family and friends (*P*<0.001). The details are given in Table 4.

**Table 4:** Risk perceptions across hcws different characteristics

	Q1		Q2		Q3		Overall risk perception
Variable	No	Yes	No	Yes	No	Yes	Mean±D
Age groups							
20–25	14 (16.3)	72 (83.7)	26 (30.2)	60 (69.8)	23 (26.7)	63 (73.3)	7.12±2.21
26–32	16 (7.0)	211 (93)	37 (16.3)	190 (83.7)	55 (24.2)	172 (75.7)	7.19±2.27
>32	11 (19)	47 (81.0)	4 (6.9)	54 (93.1)	10 (17.2)	48 (82.8)	7.72±2.09
*P* value	0.007		0.001		0.404		0.237
Sex							
Male	31 (10.8)	256 (89.2)	54 (18.8)	233 (81.2)	70 (24.3)	217 (75.7)	7.04±2.26
Female	10 (11.9)	74 (88.1)	13 (15.5)	71 (84.5)	18 (21.4)	66 (78.6)	8.01±1.97
*P* value	0.777		0.484		0.575		<0.001
Profession							
Physician	1 (11.1)	8 (88.9)	0 (0)	9 (100)	2 (22.2)	7 (77.8)	8.11±1.69
Residents Medical Officer	4 (4.8)	79 (95.2)	15 (18.1)	68 (81.9)	20 (24.1)	63 (75.9)	6.80±2.24
Medical Officer	2 (4.3)	44 (95.7)	8 (16.7)	38 (83.3)	8 (16.7)	38 (83.3)	7.37±2.64
House Officer	2 (3.8)	51 (96.2)	18 (51.4)	35 (48.6)	11 (20.8)	42 (79.2)	6.85±1.95
Pharmacist	2 (7.7)	24 (92.3)	7 (26.9)	19 (73.1)	9 (34.6)	17 (65.4)	7.35±2.37
Nurses	26 (21.3)	96 (78.7)	15 (12.3)	107 (87.7)	23 (18.9)	99 (81.1)	7.68±1.97
Medical Technician	4 (12.5)	28 (87.5)	4 (12.5)	28 (87.5)	15 (46.9)	17 (53.1)	7.06±2.76
*P* value	0.001		0.015		0.029		0.052
Job experience (years)							
≤2	17 (9.9)	154 (90.1)	43 (25.1)	128 (74.9)	51 (29.8)	120 (70.2)	6.93±2.22
3–5	9 (7.8)	107 (92.2)	10 (8.6)	106 (91.4)	26 (22.4)	90 (77.6)	7.18±2.43
6–10	15 (17.9)	69 (82.1)	14 (16.7)	70 (83.3)	11 (13.1)	73 (86.9)	8.04±177
*P* value	0.065		0.002		0.012		0.001
Frontline HCW							
Yes	29 (12.3)	207 (87.7)	41 (17.4)	195 (82.6)	34 (14.4)	202 (85.6)	7.50±2.10
No	12 (8.9)	123 (91.1)	26 (19.3)	109 (80.7)	54 (40)	81 (60)	6.84±2.40
*P* value	0.390		0.675		<0.001		0.011
Source of information regarding COVID-19							
Social media	22 (12.4)	156 (87.6)	29 (16.3)	149 (83.7)	34 (19.1)	144 (80.9)	7.36±2.11
*P* value	0.508		0.420		0.051		0.451
Print and electronic media	22 (16.8)	109 (83.2)	22 (16.8)	109 (83.2)	24 (18.3)	107 (81.7)	7.52±1.95
*P* value	0.041		0.674		0.075		0.148
Official Govt websites, WHO, CDC, Upto date etc	18 (9.0)	182 (91.0)	36 (18.0)	164 (82.0)	41 (20.5)	159 (79.5)	7.38±2.16
*P* value	0.187		1.000		0.142		0.262
Family and friends	24 (20.9)	91 (79.1)	21 (18.3)	94 (81.3)	16 (13.9)	99 (86.1)	8.01±1.85
*P* value	<0.001		1.00		0.003		<0.001
More than one source	31 (11.5)	238 (88.5)	51 (19.0)	218 (81.0)	58 (21.6)	211 (78.4)	7.28±2.18
*P* value	0.714		0.546		0.133		0.729

Q1: COVID-19 is a significant public health problem in Pakistan, Q2; Do you feel distressed and panicked regarding COVID-19 infection in Pakistan, Q3: There is a high chance for you to be infected, Q4; Overall Perceived Risk of COVID-19 [Visual Analogue Scale].

HCW, healthcare worker.

Out of 287 participants (77.36% male, mean age 29.10±5.32 years, 61.2% in the 26–32 age group), nurses formed the largest group (32.9%), and 46% had less than or equal to 2 years of job experience (Table 1). The COVID-19 knowledge score was 85.14±10.82, with a statistically significant difference between male and female HCWs (*P*=0.032) and among age groups (≥32 years: 87.59±7.33, *P*=0.021). Job experience of 3–5 years showed better knowledge (86.38±7.38, *P* value not significant). HCWs relying on family and friends had a lower mean score (82.26±15.39, *P*=0.016) (Table 3). Regarding risk perception, 88.9% agreed COVID-19 is a significant public health problem. The mean overall risk perception score was 7.26±2.23, with significant differences between males and females (*P*<0.05), various job experience categories, and frontline versus non-frontline HCWs. Those discussing COVID-19 with family and friends showed higher risk scores (*P*<0.001) (Table 4).

## Discussion

Khyber Pakhtunkhwa’s healthcare system comprises a network of public and private hospitals. However, challenges such as limited resources, particularly in rural areas, and occasional strain on healthcare facilities during health crises, contribute to the complex landscape.

HCWs in Khyber Pakhtunkhwa have access to training programs and resources provided by the provincial health department. Availability of personal protective equipment (PPE) varies, with urban centres generally having better access compared to remote areas. Geographic factors pose challenges, especially in reaching remote communities. Additionally, the healthcare system sometimes faces shortages in critical supplies during health emergencies, impacting the preparedness of healthcare workers.

The global spread of the COVID-19 outbreak has caused outrage among healthcare systems and staff around the world, as well as increased fear and anxiety. The key elements that need to be taken into account in this regard are knowledge and risk perception. Our study is perhaps the first of its kind to assess the knowledge and risk perception regarding COVID-19 in HCWs in Peshawar, Pakistan.

Sufficient knowledge and efficient precautionary measures create awareness among patients and the general population. Lack of appropriate knowledge among HCWs and deficiencies in the practice can be one of the reasons for the spread of the virus^26^.

The PMS of COVID-19 knowledge in this survey was 85.14±10.82, which is greater than the earlier studies performed on MERS in HCWs and slightly lower than a similar report in South Korea^27,28^. However, a study by Zhou *et al.* ^29^, reported that 89% of the HCWs demonstrated sufficient knowledge and similarly a recent local study also reported good knowledge of HCWs^30^. Shi *et al.* ^31^, and Giao *et al.* ^32^, reported that 89.51% and 88.4% of HCWs have good knowledge respectively. In contrast, a similar study of nurses found that 56.5% have sufficient knowledge in terms of disease symptoms, transmission, and treatment of COVID-19^33^. Knowledge is a prerequisite for formulating prevention beliefs, shaping positive attitudes, and encouraging positive behaviours. Moreover, individual awareness and attitude towards disease also have an impact on the usefulness of their coping mechanisms and behaviours to a certain extent.

In this study, 200 (53.9%) HCWs used official websites like WHO, CDC, UpTodate and research studies etc. for getting information on COVID-19 with 269 (72. 51%) who used more than one source. Nearly 48% used social media as an information tool. These results contradict those of Giao and colleagues, who found that 91.1% of HCWs use social media as a primary source of knowledge about COVID-19^32^. Going in the same direction another study among HCWs reported more than 60% using social media for seeking information related to COVID-19^34^. It is also different from a local study which shows that 87.68% of HCWs used social media as their primary source of information and only 23.19% obtained information from educational seminars and workshops. Besides a disease pandemic, an infodemic regarding COVID-19 might lead to xenophobic attitudes in different parts of the world^35^. Furthermore, there is an abundance of misleading and unverified content circulating on the internet that has the potential to misguide HCWs. To find information, HCWs must carefully review COVID-19 information sources and use accurate and relevant material^34^. With time, updated and authentic information is being uploaded to the official sites and in the same way new articles are being published in different scientific journals. HCWs should thoroughly and efficiently use sources of knowledge that are credible and well-referenced, to keep themselves updated about the pandemic.

A high score in the knowledge component was recorded by HCWs in our study. The majority of the HCWs (98.9%) were aware that hand hygiene, covering nose, and mouth while coughing and sneezing and avoiding contact with sick people can help in the prevention of SARS-COV-2 transmission. About 99% were aware of the common signs and symptoms. The majority of the HCWs 96.23% were aware that there is no definitive treatment for the virus. Likewise, the majority thought that people who came in contact with infected patients should be isolated immediately for 14 days. The findings of this study give HCWs confidence in their awareness of COVID-19 symptoms, management protocol of the disease, spreading and preventive actions. This is of utmost importance in the present scenario when there is no vaccine and definite antiviral therapy.

Our study findings suggest a lack of knowledge amongst HCWs regarding the incubation period of COVID-19, the use of N95 masks and the approximate distance through which the virus can travel and transmit the infection. Owing to the devastating nature of the pandemic and despite the provision of a great number of resources to educate HCWs, such findings indicate a great concern. The possible assumption could be the discussion happening among HCWs is more definitely regarding the symptoms and management of the disease compared to other aspects like the incubation period, the worth of the N95 mask and the distance they think that the coronavirus can travel through the air to transmit the infection from one person to another. These aspects of COVID-19 must therefore be clarified and discussed with HCWs so that they can inform people about how to combat this global public health crisis. Hence, our findings are disappointing and it is suggested that the national health authorities and medical institutions should encourage HCWs to assimilate current and evidence-based knowledge related to COVID-19.

The HCWs’ knowledge about COVID-19 has the highest mean score (87.59±7.33) in age older than or equal to 32 years compared to the age groups 20–25 years (81.09±17.05) and 26–32 years (86.05±7.77) and the differences were statistically significant (*P*=0.021). A study by Aldohyan *et al.* ^36^, while evaluating MERS-CoV educational programs and knowledge transfer showed that HCWs more than 40 years old had secured the highest scores compared to other age groups (*P*=0.005). Our results are opposite to the findings, which showed that age was significantly associated with good knowledge, as 95.5% of HCWs under 30 years of age had sound knowledge relative to other age groups’ HCWs^30^.

Male HCWs scored high (PMS 85.62±1.08; *P*=0.032) compared to the females (PMS 83.49±9.79). This is not in line with the recent studies published both internationally and locally where knowledge score was not significantly different between males and females^26,30^. Likewise, an earlier study on MERS also reported contradictory results compared to our study^36^.

In our study physicians had higher knowledge scores compared to the others in the group, but the differences were insignificant. Bhagavathula *et al.* also reported the same results^34^ On the other hand Giao *et al* ^32^. observed that pharmacists have higher knowledge compared to other HCWs regarding COVID-19 (*P*=0.015). Moreover, Khan and colleagues and Albarrak and colleagues also found that allied healthcare workers including pharmacists have a greater understanding of MERS compared to physicians^27,37^. Probable assumptions to this could be the rapidly evolving role of pharmacists from dispensing tables to patient beds and their active part in improving patient care outcomes in COVID-19. Furthermore, the findings support a collaborative approach among various HCWs when it comes to clinical decision-making.

In addition, HCWs with 3–5 years of work experience had better COVID-19 knowledge (PMS 86.38±7.38) compared to HCWs with 2 years of experience (PMS 84.05±13.36) and 6–10 years of experience (PMS 85.63±8.75), respectively. Still, the mean score was not significantly different. A study conducted on nurses in Iran shows that the overall score of awareness was not affected by age and education level, and it was not significantly different among nurses irrespective of work experience^33^.

HCWs 93.1% with the age group older than 32 years (*P*=0.001), all physicians (*P*=0.015) and participants with job experience of 3–5 years (*P*=0.002) thought to be preoccupied/concerned and terrified regarding the COVID-19 epidemic in Pakistan. About 81.1% of the nurses (*P*=0.029), 202 (54.4%) frontline HCWs and those with a job experience of 6–10 years 86.9% (*P*=0.012) were paranoid with the contemplation of contracting COVID-19.

Huang and Zhao found that HCWs had higher levels of anxiety than other staff in a survey^38^. During the MERS epidemic, a study reported a high level of anxiety in medical students in Saudi Arabia^39^. The fear of being infected, the difficulty in controlling the disease and the lack of medical facilities around the country are the most likely causes of such high anxiety. A recent study by Minghe Zhou^29^, reported that being a paramedic staff is highly associated with a level of fear of COVID-19. A recent study in Iran described a moderate level of risk perception in terms of contracting COVID-19 infection more easily than others^26^. Frontline HCWs who come into direct contact with a diagnosed or suspected by various means have a high risk of getting an infection^29^.

Furthermore, overall HCWs’ perceived risk of COVID-19 was significantly (*P*<0.001) different among males (7.04±2.26) and females (8.01±1.97). These findings are the opposite of the findings from a study done in Iran involving medical students reporting mean risk perception lower (*P*<0.001) among females (3.72±1.66) compared to males (4.60±1.8)^26^. Its reason could be an increased awareness among males which justifies the high mean score of knowledge of males compared to the females. Regarding the job experience of HCWs, the VAS score did differ significantly with job experience of 6–10 years securing a higher value (8.04±177) than other groups. It could be due to high job experience and greater judgmental insight.

Similarly, frontline HCWs perceived high overall risk compared to non-front liners. Frontline HCWs’ high perception is justified due to direct contact and monitoring of the overall situation. Furthermore, an overall perceived risk score was higher for those who had received the information or discussed COVID-19-related topics with their family and friends (*P*<0.001).

The universal combat of the pandemic triggered by severe acute respiratory syndrome coronavirus-2 relies on successfully implementing a rapid immunization program, with optimum vaccine distribution, coverage and efficacy^40^.

This includes COVID-19 vaccines which aid in hindering virus spread and protect personal health^41^.

The vaccine-induced immune responses may be elicited against all the current viral mutations; however, over time, as the mutated lineages emerge beyond the present available categorization as per the evolutionary advantage conferred to SARS-CoV-2, there may be a need to modify the vaccines in future^42^.

Adopting mass vaccination against COVID-19 vaccine has become a key measure for countries to fight against the epidemic^43^. In addition, the development and approval of intra-nasal vaccines against COVID-19 have turned out to be a global game changer^44^. Inspite of such significant progress which has been achieved in preventing and treating SARS-CoV-2, acute infection remains a subject of ongoing research^45,46^.

### Strengths

#### Clear objectives

The study aims to assess the knowledge and risk perception of COVID-19 among HCWs in Khyber Pakhtunkhwa, Pakistan. The objectives are well-defined and provide a clear focus for the research.

#### Adequate sample size

The study includes a sufficient sample size of HCWs from major tertiary care facilities in Khyber Pakhtunkhwa, which enhances the generalizability of the findings.

#### Comprehensive data collection

The use of a web-based online questionnaire comprising 26 items allows for the collection of a wide range of information related to knowledge and risk perception of COVID-19 among HCWs.

#### Statistical analysis

The study employs appropriate statistical analysis techniques to analyze the data, including percentage mean scores and comparisons between different groups. This adds rigour to the study’s findings.

#### Valuable findings

The study reveals that HCWs in Khyber Pakhtunkhwa generally have good knowledge about COVID-19, but their risk perception is high, which may impact their self-protective behaviour and mental health. These findings are important for addressing the concerns and needs of HCWs during the pandemic.

#### Validation of findings

While self-reported data was primarily used, we cross-referenced responses with hospital records for certain aspects, such as COVID-19 cases among healthcare workers. This validation method aimed to enhance the reliability of our findings.

### Limitations

The first limitation of this study is that it was confined to those respondents who had access to the Internet. Secondly, this study represents the HCWs of a single province and its result cannot be generalized to all HCWs across the country. A large-scale study among HCWs on a national level could more truly be representative of our aims and objectives. Thirdly, the data provided in our analysis is based on self-reporting and the recall biases cannot be ruled out. Fourthly, our study has not addressed attitudes related to the COVID-19 pandemic. This section could be incorporated into future studies. The data collected through a self-reported online questionnaire may be subject to response bias and may not accurately represent the actual knowledge and risk perception of HCWs.

## Conclusion

Despite some gaps, HCWs demonstrated good COVID-19-related knowledge. We found in our study that risk perception regarding COVID-19 was high. As the situation continues to worsen, greater efforts through educational intervention programs and campaigns are required for HCWs to update them with the knowledge, transmission mode, Mode, isolation time and treatment protocols. HCWs should also be educated about the risk of personally acquiring the infection and passing it on to family members. As frontline workers are the most vulnerable, stakeholders must consider this issue more promptly and seriously.

### Implications and recommendations

The study underscores the need for targeted interventions to address knowledge gaps and enhance risk perception among healthcare workers. Policymakers should consider tailored training programs and resource allocation to better support healthcare workers. Based on our findings, we recommend developing specialized training modules focusing on critical aspects of COVID-19, such as transmission dynamics and proper use of PPE. Additionally, periodic updates and refresher courses can help healthcare workers stay abreast of evolving information.

## Ethics approval

The study protocol was approved by the ethics and research committee in Hayatabad Medical Complex, Peshawar, Pakistan. (Reference #: 1509–2020). Trail registry Number: College of Physical Medicine and Rehabilitation. Number: CPMR /PCP/29421.

## Consent to participate

Written informed consent was obtained from the patients for publication and any accompanying images. A copy of the written consent is available for review by the Editor-in-Chief of this journal on request.

## Sources of funding

The authors received no financial support for the research, authorship, and /or publication of this article.

## Author contribution

I.A.: conception, data analysis, addition of contents to the initial version, review and editing. Z.H., A.A.: drafting of initial version and language editing. A.R.U., M.N.K.W. and A.M., N.F., S.Z.A.S. and W.A.: data collection, initial drafting review, organisation. M.I.K., I.U., A.A., A.H.. A.A., L.H., G.E.M.A. and K.A.: added ideas and content to the first version, data collection and review. All authors read and approved the final version.

## Conflicts of interest disclosure

The authors declared no potential conflicts of interest concerning the research, authorship, and /or publication of this article.

## Research registration unique identifying number (UIN)

1. Name of the registry: Not applicable.
2. Unique Identifying number or registration ID: Not applicable.
3. Hyperlink to your specific registration (must be publicly accessible and will be checked): Not applicable.

## Guarantor

Khabab Abbasher Hussien Mohamed Ahmed.

## Availability of data and material

The data that support the findings of this survey is available on request from the corresponding author.

## Provenance and peer-review

Not commissioned, externally peer-reviewed.
